# Identification of molecular subtypes and prognostic signatures based on transient receptor potential channel-related genes to predict the prognostic risk of hepatocellular carcinoma: A review

**DOI:** 10.1097/MD.0000000000033228

**Published:** 2023-03-10

**Authors:** Dongyang Wu, Qingshan Cai, Dong Liu, Ganggang Zuo, Shudong Li, Liyou Liu, Jianxing Zheng

**Affiliations:** a Department of Hepatobiliary Surgery, Tangshan Central Hospital, Tangshan City, Hebei Province, China.

**Keywords:** hepatocellular carcinoma, molecular subtypes, prognostic model, transient receptor potential channel

## Abstract

Abnormal transient receptor potential (TRP) channel function interferes with intracellular calcium-based signaling and causes malignant phenotypes. However, the effects of TRP channel-related genes on hepatocellular carcinoma (HCC) remain unclear. This study aimed to identify HCC molecular subtypes and prognostic signatures based on TRP channel-related genes to predict prognostic risks. Unsupervised hierarchical clustering was applied to identify HCC molecular subtypes using the expression data of TRP channel-related genes. This was followed by a comparison of the clinical and immune microenvironment characteristics between the resulting subtypes. After screening for differentially expressed genes among subtypes, prognostic signatures were identified to construct risk score-based prognostic and nomogram models and predict HCC survival. Finally, tumor drug sensitivities were predicted and compared between the risk groups. Sixteen TRP channel-related genes that were differentially expressed between HCC and non-tumorous tissues were used to identify 2 subtypes. Cluster 1 had higher TRP scores, better survival status, and lower levels of clinical malignancy. Immune-related analyses also revealed higher infiltration of M1 macrophages and higher immune and stromal scores in Cluster 1 than in Cluster 2. After screening differentially expressed genes between subtypes, 6 prognostic signatures were identified to construct prognostic and nomogram models. The potential of these models to assess the prognostic risk of HCC was further validated. Furthermore, Cluster 1 was more distributed in the low-risk group, with higher drug sensitivities. Two HCC subtypes were identified, of which Cluster 1 was associated with a favorable prognosis. Prognostic signatures related to TRP channel genes and molecular subtypes can be used to predict HCC risk.

**Key Points**

1. Two molecular subtypes of hepatocellular carcinoma were identified based on 16 transient receptor potential channel-related genes.
2. Cluster 1 was associated with a favorable prognosis and was more prevalent in the low-risk group.
3. Six prognostic signatures were used to calculate risk scores, which were defined as independent prognostic risk factors.
4. Risk score-based prognostic models can be used to stratify and predict the risk of HCC.

## 1. Introduction

Hepatocellular carcinoma (HCC) is a primary malignant hepatocyte tumor, accounting for approximately 80% of liver cancer cases worldwide.^[1,2]^ According to annual projections by the World Health Organization, >1 million patients are expected to die of liver cancer by 2030.^[3]^ The progression of cirrhosis is the biggest risk factor for HCC, and it is estimated that 90% of patients with underlying cirrhosis will eventually develop HCC.^[4]^ Previous studies have demonstrated the beneficial chemopreventive effect of insulin-sensitizing drugs or statins against HCC occurrence.^[5,6]^ Surgical resection, radiofrequency ablation, and liver transplantation are the first therapy choices for early HCC, with a 5-year survival rate of 70%.^[7,8]^ During the last 15 years several aspects of HCC scenario have changed, as well as its management.^[9,10]^ However, these therapies are ineffective in advanced stages (such as in metastatic HCC) where tumor resection is impossible, resulting in a 5-year survival rate as low as 2.5%.^[11]^ A series of genetic events contribute to the pathogenesis and progression of HCC, 25% of which result from inducible mutations. These molecular alterations determine the abnormal proliferation of tumor cells and their subsequent invasion.^[12]^ Therefore, biomarkers characterizing molecular genetic mechanisms are essential for assessing disease prognosis and predicting treatment responses.

Transient receptor potential (TRP) channels are a class of cation channels that perform signal transduction upon activation by changing the membrane potential or intracellular calcium (Ca2^+^) concentration^[13]^ Ca^2+^ signaling is crucial for regulating key cellular events, including gene transcription, movement and contraction, energy production, and channel control.^[14]^ The mammalian TRP superfamily consists of 28 nonselective cation-permeable channels that can be divided into 6 subfamilies based on sequence homology.^[15]^ In cancer, mutations in TRP channel-encoding genes generate TRP channels with abnormal functions that interfere with normal intracellular Ca^2+^ distribution patterns, resulting in the dysregulation of downstream effectors and enhanced cancer-specific pathological features.^[16]^ For example, the expression of *TRPV2* has been implicated in the drug-induced cytotoxicity and stemness of HCC.^[17,18]^ In addition, the expression of *TRPV2* increases as liver disease progresses from normal liver to chronic hepatitis and then to cirrhosis and may serve as a prognostic marker for patients with HCC.^[19]^ Another TRP superfamily gene, *TRPC7*, is highly expressed in viral hepatitis B-associated HCC and may be a potential therapeutic target or diagnostic marker.^[20]^ Therefore, genes encoding TRP channels may play crucial roles in the progression of HCC. However, the understanding of the role of all 28 channel-related genes is still incomplete, and systematic studies on their impact on the prognosis of HCC are lacking.

To this end, we analyzed 28 TRP channel genes from public expression data and established HCC molecular subtypes and prognostic signatures to stratify and predict the prognostic risk of HCC. We further constructed and validated a risk prognosis model and analyzed the resulting prognostic values reported for the immune microenvironment characteristics and drug sensitivity of TRP channel genes to comprehensively elucidate the potential role of these genes in HCC.

## 2. Methods

### 2.1. Data sourcing and preprocessing

Standardized gene expression profiles and clinical follow-up data of patients with HCC were obtained from The Cancer Genome Atlas (TCGA) database. After removing samples with no overall survival data, 365 HCC samples with prognostic information were retained. The microarray dataset GSE14520^[21]^ downloaded from the Gene Expression Omnibus database was defined as an independent external validation cohort. GSE14520 is present on the sequencing platform of the GPL3921 [HT_HG-U133A] Affymetrix HT Human Genome U133A Array and contains 221 HCC samples with valid prognostic data.

### 2.2. Subtype identification based on TRP channel genes

Data on the 28 TRP channel genes used in this study are provided in a relevant published article.^[22]^ We compared expression levels between HCC and non-tumorous samples and used Pearson correlation analysis to assess potential associations. We identified the corresponding HCC subtypes based on TRP channel-related genes with significant differences in expression, which were subjected to unsupervised hierarchical clustering using R 3.6.1 ConsensusClusterPlus Version 1.54.0 (http://www.bioconductor.org/packages/release/bioc/html/ConsensusClusterPlus.html). The optimal *k* value ranged from 2 to 6. The TRP score in each sample was determined by enrichment scores computed by the gene set variation analysis algorithm using the R gene set variation analysis package version 1.36.2 (http://bioconductor.org/packages/release/bioc/html/GSVA.html)^[23]^ and subsequently compared among subtypes using the Wilcoxon test to verify the rationality of HCC subtypes.

### 2.3. Association analysis between HCC subtypes and clinical features

To evaluate the correlation between prognostic survival and different HCC subtypes, Kaplan–Meier (KM) curves were generated using the R survival package Version 2.41-1 (http://bioconductor.org/packages/survivalr/).^[24]^ The clinical characteristics of patients with HCC (including age, sex, tumor grade, pathological stage, pathological TNM classification, and exposure to radiation therapy) were analyzed. Furthermore, the relationships between subtypes and these clinical features were assessed using the chi-square test, and statistical significance was set at *P* < .05.

### 2.4. Comparison of immune microenvironment among subtypes

In this study, we applied 2 algorithms to evaluate the immune microenvironment status of HCC samples and compared immune infiltration among different HCC subtypes using the Wilcoxon signed-rank test. For immune infiltration analysis, CIBERSORT (httRiskscore://cibersort.stanford.edu/index.php)^[25]^ was used to compute the proportions of 22 types of immune cells, whereas ESTIMATE^[26]^ was used to estimate the stromal and immune scores of the tumor samples.

### 2.5. Screening of differentially expressed genes (DEGs) among HCC subtypes

To observe the possible existence of different molecular mechanisms underlying HCC subtypes, DEGs between these subtypes were screened using linear regression and empirical Bayesian methods provided by the limma package, version 3.10.3 (http://www.bioconductor.org/packages/2.9/bioc/html/limma.html).^[27]^ The obtained *P* values were adjusted for multiple tests using the Benjamini–Hochberg method, and genes characterized by an adjusted *P* value < 0.05 and |log_2_fold change| > 1 were considered to have significant differential expression.

### 2.6. Identification of prognostic signatures

Based on the obtained DEGs, univariate Cox regression analysis was used to screen the expression levels of genes significantly associated with survival with a set threshold of *P* < .01. The least absolute shrinkage and selection operator algorithm was then used to define an optimal lambda value, followed by subsequent assessment of prognostic signatures (10-fold cross-validation) using the R package for the LARS algorithm version 1.2 (https://cran.r-project.org/web/packages/lars/index.html).

### 2.7. Generation and validation of the risk score-based prognostic model

Furthermore, the prognostic gene signatures were subjected to stepwise Cox regression analysis using the R survminer package version 0.4.9 (https://cran.rstudio.com/web/packages/survminer/index.html) to establish a risk score-based prognostic model. The risk score was calculated using the following formula:


$$\begin{array}{l} Risk\;score=\;h_{0}\left(t\right)\;*\;exp\;(\beta_{1}X_{1}+\;\beta_{2}X_{2}+...\;+\beta_{n}X_{n}) \end{array}$$

where *β* is the regression coefficient, h_0_(t) is the baseline risk function, and h(t, X) is the risk function associated with X (covariate) at time t. We further calculated the risk scores of each sample in TCGA and GSE14520 cohorts and grouped the samples according to their median values. The samples were divided into high-risk prognosis (HRP) and low-risk prognosis (LRP) groups, and a KM curve was created to estimate the difference in the actual prognosis.

### 2.8. Analysis of prognostic independence to construct a nomogram model

We assessed clinical characteristics using univariate and multivariate Cox regression analyses to screen for independent prognostic factors at a threshold value of *P* < .05. The R.rms package version 5.1-2 (https://cran.r-project.org/web/packages/rms/index.html)^[28]^ was used to construct a nomogram model to predict the survival probability of patients with HCC.

### 2.9. Drug sensitivity analysis

The Genomics of Drug Sensitivity in Cancer database was used to estimate the sensitivity of each sample to chemotherapy drugs, followed by quantification of 50% inhibitory concentration (IC50) values using the R pRRophetic package (https://github.com/paulgeeleher/pRRophetic).^[29]^ The IC50 values of 138 chemotherapy drugs were compared between the HRP and LRP groups using the Wilcoxon signed-rank test.

### 2.10. Association analysis of prognostic risk and HCC subtype

To observe the relationship between HCC subtypes and risk groups, we compared the distribution differences of subtypes between the HRP and LRP groups. The results were visualized using a bar chart obtained with the R. galluvial package version 0.12.3 (https://CRAN.R-project.org/package=ggalluvial).^[30]^

## 3. Results

### 3.1. Two HCC subtypes were identified based on the analysis of TRP channel-related genes

**Figure 1.**
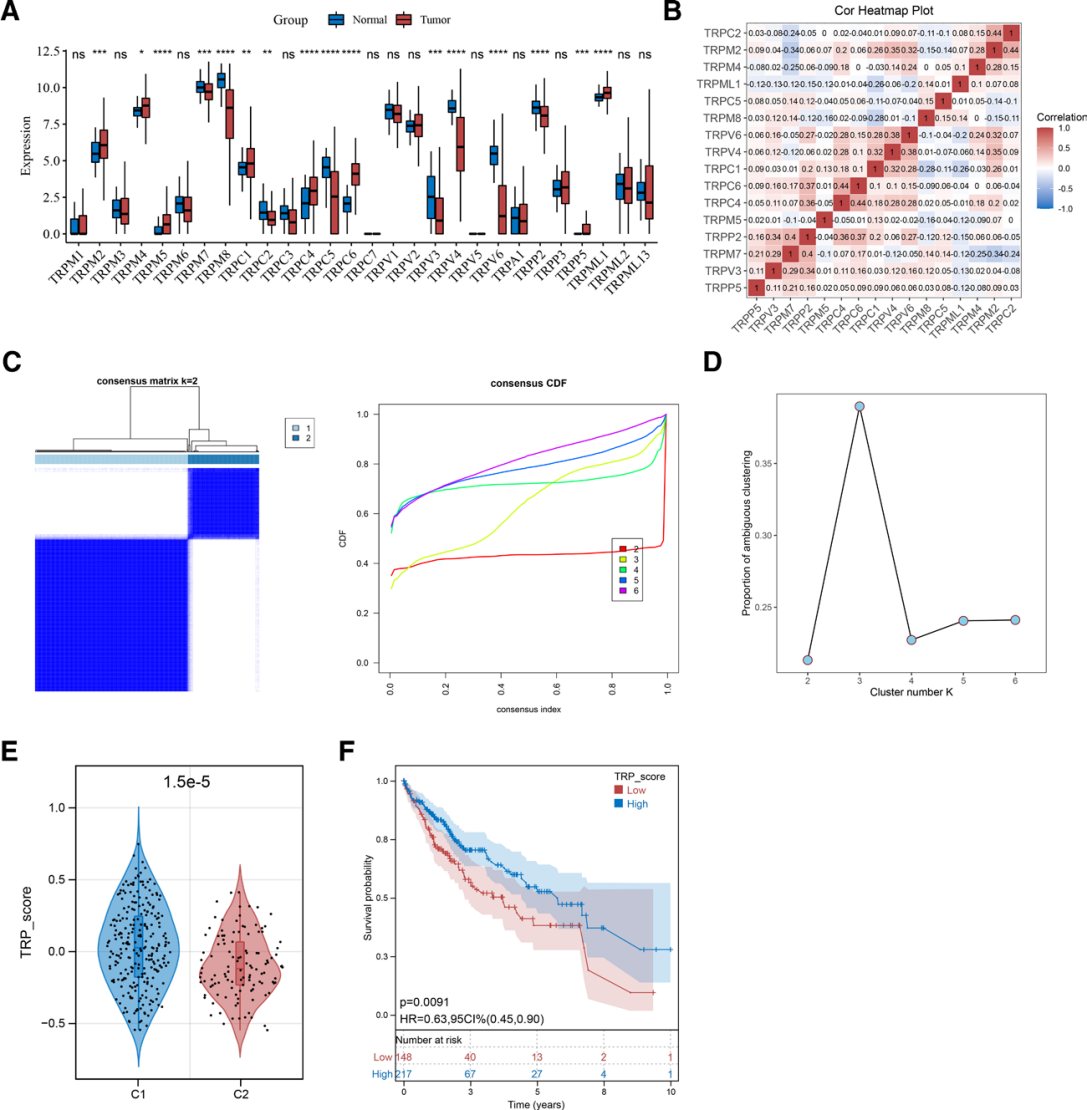
Two HCC subtypes associated with prognosis were identified based on 16 key TRP channel-related genes. (A) Differences in the expression of 28 TRP channel-related genes between HCC and normal tissue samples. *P < .05, **P < .01, ***P < .001, ****P < .0001. (B) Correlation between the expression of 16 key TRP channel-related genes. (C) Consensus clustering cumulative distribution function with k = 2–6. (D) Verification graph based on the proportion of ambiguous clusters. (E) Difference in TRP score between the 2 subtypes. (F) KM curve showing the difference in survival between patients with high and low TRP scores. HCC = hepatocellular carcinoma, KM = Kaplan–Meier, TRP = transient receptor potential.

To analyze the expression patterns of the 28 TRP channel-related genes, their expression profiles were compared between HCC and non-tumorous samples. Sixteen TRP channel-related genes showed significant differences in expression levels between the 2 groups (Fig. 1A). Correlations between the expression of these genes were also analyzed; the results suggested that *TRPV4* and *TRPV6* had the strongest positive correlation, whereas *TRPM2* and *TRPM7* had the strongest negative correlation (Fig. 1B). The 16 key TRP-related genes were then incorporated into the unsupervised cluster analysis, and the optimal subtype number was defined as k = 2 (Fig. 1C). The proportion of ambiguous clustering was then determined to verify the stability of the clustering results, and the optimal value of k = 2 was confirmed (Fig. 1D). The TRP score of each sample was calculated and compared between the 2 clusters. The TRP score of Cluster 1 was significantly higher than that of Cluster 2 (Fig. 1E). Survival analysis also indicated a poor prognosis in patients with a low TRP score (Fig. 1F). These results highlight that a higher TRP value, as exhibited by Cluster 1, may be a favorable prognostic factor.

### 3.2. Differences in clinical features and prognosis between subtypes

**Figure 2.**
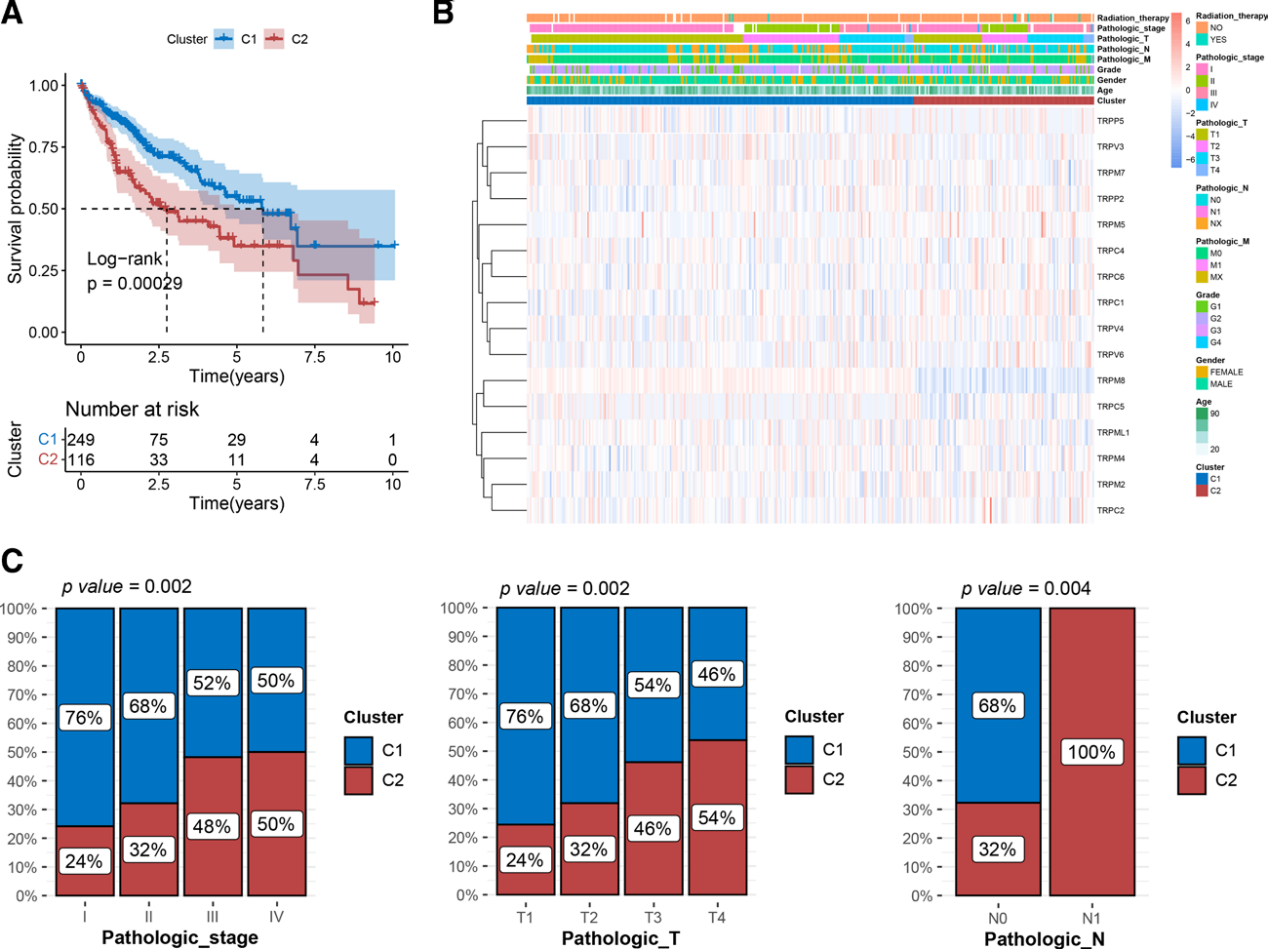
Differences in survival and the distribution of clinical characteristics between cluster 1 and cluster 2. (A) The KM curve shows a significant difference in survival probability between the 2 clusters. (B) Heatmap reveals the expression patterns of 16 key TRP genes reported to different clinical features. (C) The distribution proportions of clusters 1 and 2 varied significantly reported to clinical characteristics. KM = Kaplan–Meier, TRP = transient receptor potential.

To further explore the relationship between clinical characteristics and HCC subtypes, we conducted a survival analysis to compare the survival differences between Clusters 1 and 2 (Fig. 2A). The KM curve suggested a better prognosis for patients in Cluster 1, which is consistent with previous results. In addition, the distribution of expression of the 16 key TRP-related genes associated with different clinical features was visualized using a heatmap (Fig. 2B). Comparison among subtypes revealed that the distribution of Cluster 2 was significantly associated with the later period of pathologic T, pathologic N, and pathologic stage (Fig. 2C). This suggests that Cluster 2 might possess a prognostic risk value, indicating the deterioration of clinical manifestations.

### 3.3. Analysis of immune microenvironment characteristics based on HCC subtype

**Figure 3.**
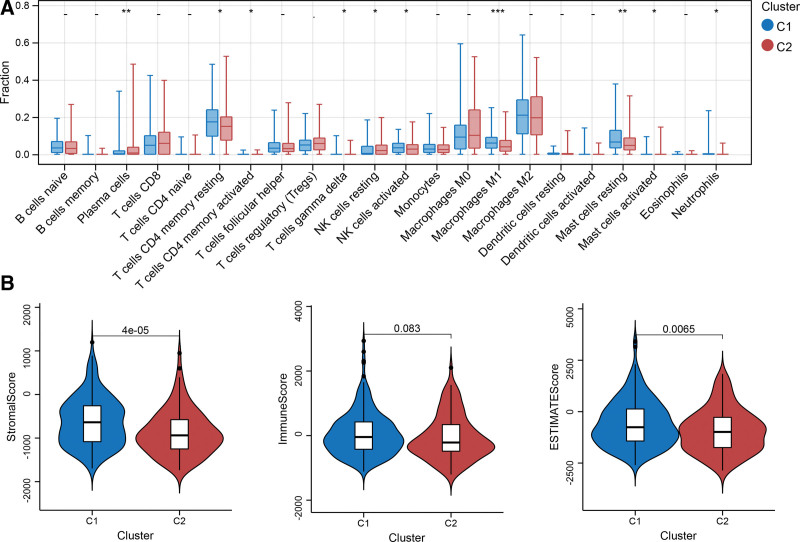
Comparison of immune cell infiltration between cluster 1 and cluster 2 using CIBERSORT and ESTIMATE algorithms. (A) Infiltration differences in 22 types of immune cells between the 2 clusters were evaluated using the CIBERSORT algorithm. *P < .05, **P < .01, ***P < .001. (B) The violin plot depicts the difference in stromal and immune scores between clusters 1 and 2, which were calculated using the ESTIMATE algorithm.

Based on the expression matrix of HCC samples, the CIBERSORT algorithm was employed to calculate the fractions of immune cells in each sample. Using a threshold value of *P* < .05, 10 types of immune cells were found to infiltrate significantly differently between Clusters 1 and 2 (Fig. 3A). Among these, M1 and M2 macrophages exhibited the most significant difference between the 2 groups; their infiltration levels were higher in Cluster 1 than in Cluster 2. The ESTIMATE algorithm was used to compute the stromal and immune scores for each sample. Compared to Cluster 2, Cluster 1 presented higher immune and stromal scores (Fig. 3B), which also indicated a higher level of non-tumor cell infiltration.

### 3.4. Optimal prognostic signatures were screened based on DEGs between HCC subtypes

**Figure 4.**
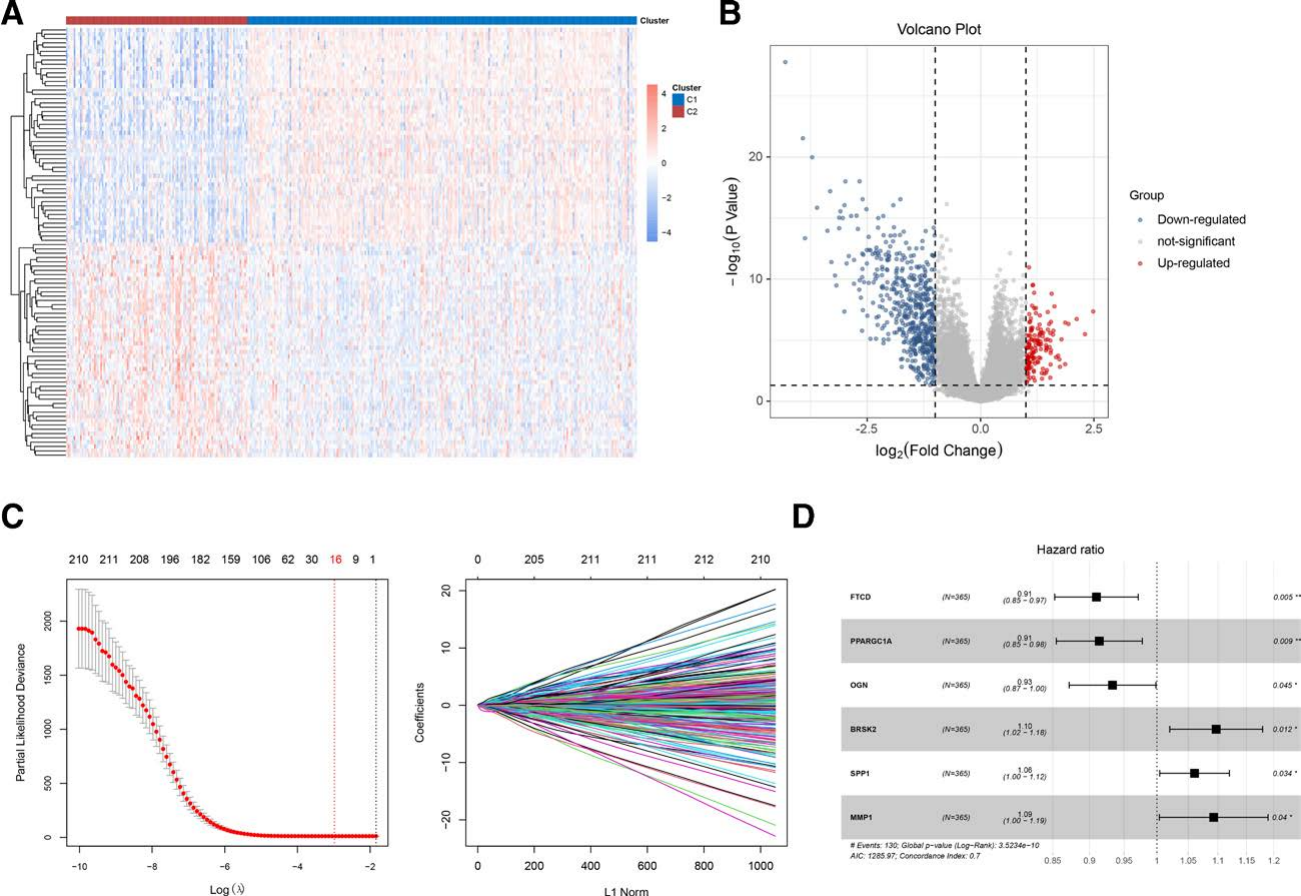
Screening of prognostic signatures for model construction based on differentially expressed genes (DEGs) between cluster 1 and cluster 2. (A) Heatmap showing the expression patterns of 678 DEGs between clusters 1 and 2. (B) The volcano plot exhibits 148 upregulated and 530 downregulated DEGs, with screening standards of adjusted *P* value < 0.05, and |log_2_FC| > 1. (C) LASSO coefficient distribution and likelihood bias. (D) The optimal gene set which included 6 prognostic signatures was identified using univariate Cox regression analysis. *P < .05, **P < .01. LASSO = least absolute shrinkage and selection operator.

To further explore the differences in molecular regulatory mechanisms among the subtypes, we performed a differential analysis of gene expression levels between Clusters 1 and 2. The patterns of the DEGs between the 2 groups are shown in Figure 4A. A total of 148 upregulated and 530 downregulated DEGs were identified according to the set thresholds (Fig. 4B). Univariate Cox regression analysis was performed to select the 212 DEGs that were significantly associated with prognosis. The least absolute shrinkage and selection operator algorithm was used to screen for optimal candidates, and 16 key genes were selected at lambda = 16 (Fig. 4C). Finally, stepwise Cox regression was used to define the optimal gene set, which constituted the basis for constructing the prognostic model. This set contained 6 prognostic signatures (matrix metalloproteinase (MMP) 1, *SPP1, BRSK2, OGN, PPARGC1A*, and *FTCD*) (Fig. 4D).

### 3.5. Prognostic model construction and efficacy verification

**Figure 5.**
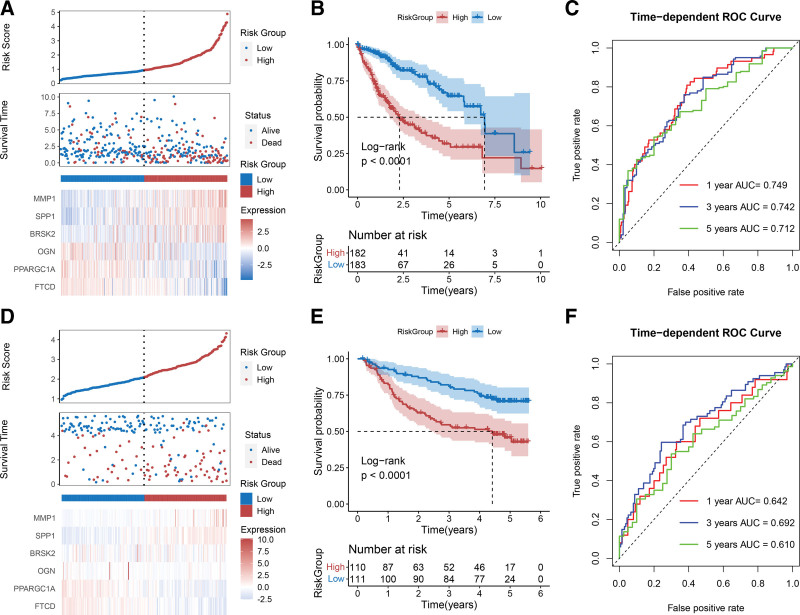
Construction and verification of a risk score-based prognostic model in training and validation cohorts. (A and D) Distribution of risk score, survival time, and expression of 6 signatures in (A) TCGA and (D) GSE14520 datasets. (B and E) KM curves show survival differences between the HRP and LRP groups in both (B) TCGA and (E) GSE14520 datasets. (C and F) ROC curves depicting the sensitivity and specificity of the (C) TCGA and (F) GSE14520 datasets in predicting the prognostic risks of HCC. HCC = hepatocellular carcinoma, HRP = high-risk prognosis, KM = Kaplan–Meier, LRP = low-risk prognosis, ROC = receiver operating characteristic, TCGA = The Cancer Genome Atlas.

Based on the regression coefficients of the 6 prognostic signatures and their levels in the TCGA dataset, a risk score-based prognostic model was constructed. The distributions of the risk score, survival time, and 6 prognostic signatures for each sample are shown in Figure 5A. These results suggest that the risk of death increases with the risk score. Furthermore, the expression of *MMP1, SPP1*, and *BRSK2* was higher in the HRP group than in the LRP group. The KM curve confirmed that the actual survival of patients in the LRP group was significantly higher than that of patients in the HRP group (Fig. 5B). A time-dependent receiver operating characteristic curve was created to assess the specificity and sensitivity of the model in predicting prognostic risks; the area under the curve (AUC) of the 1-, 3-, and 5-year prediction were 0.749, 0.742, and 0.712, respectively (Fig. 5C), which represented a high predictive performance of the model. The performance of the model based on the 6 prognostic signatures was analyzed using the GSE14520 validation dataset. The results suggested that with an increase in the risk score, patient survival time and gene expression levels tended to change (Fig. 5D). The patients in the HRP group also had a significantly lower likelihood of survival (Fig. 5E). In addition, all AUC values were >0.6, indicating that the prognostic model based on the validation cohort had the potential to predict prognostic risk (Fig. 5F).

### 3.6. Screening of independent prognostic factors to construct a nomogram survival prediction model

**Figure 6.**
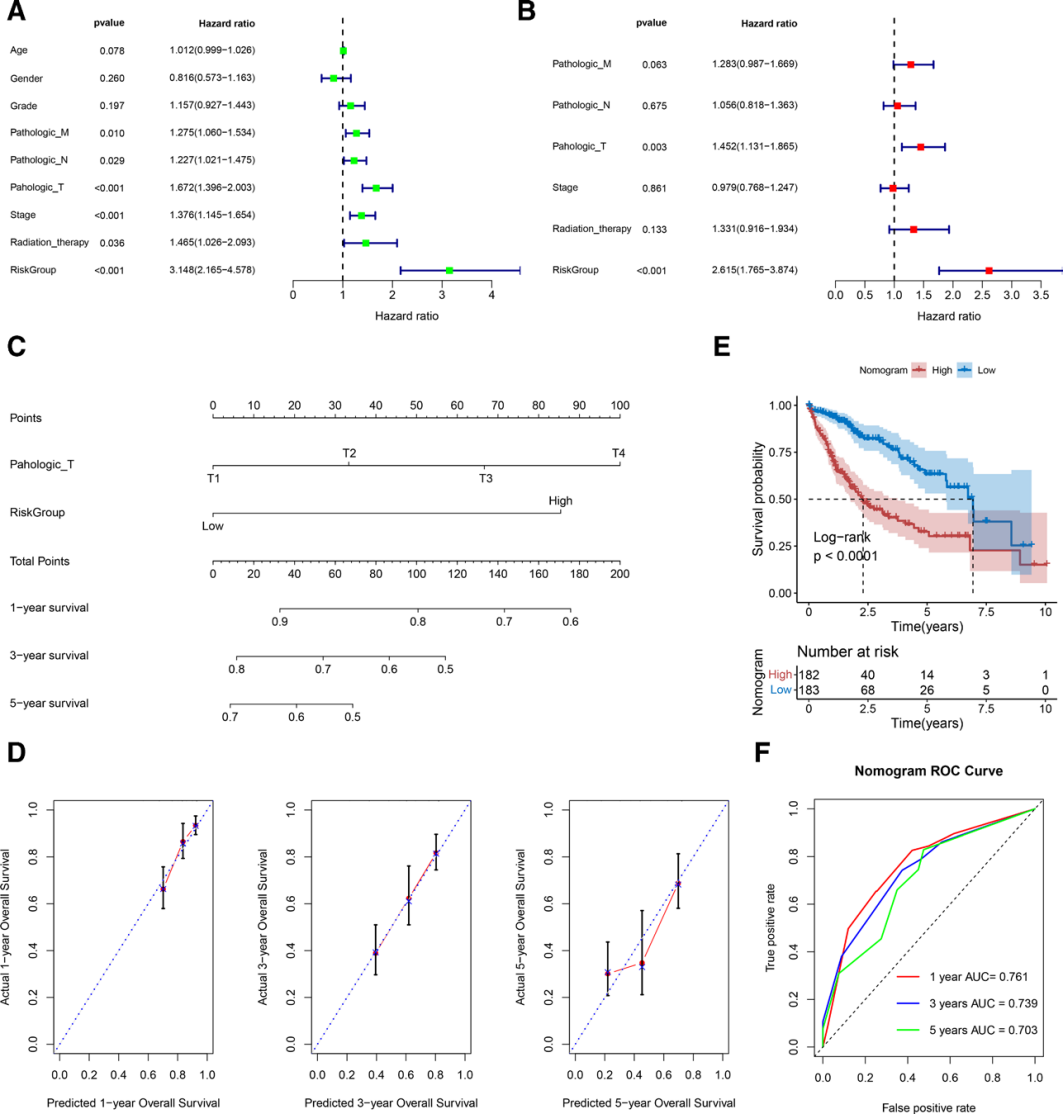
Identification of independent prognostic factors to construct a prognostic predictive nomogram model. (A and B) (A) Univariate and (B) multivariate regression analyses were performed to identify the independent prognostic factors. (C) Factors of the pathological T and risk groups were incorporated to generate a nomogram model. (D) The calibration curve illustrates consistency between the actual and predicted values of the nomogram model. (E) The KM curve indicates a significant difference in survival between patients with high and low prognostic risk as predicted by the nomogram model. The 1-, 3-, and 5-year survival ROC curves confirmed the predictive capability of the nomogram model. KM = Kaplan–Meier, ROC = receiver operating characteristic.

We applied univariate Cox regression analysis for the clinical characteristics of all HCC samples in the TCGA dataset and selected factors with *P* < .05 to further conduct the multivariate Cox regression analysis (Fig. 6A and B). Then, the factors “pathological T” and “risk groups” were identified with prognostic independence to establish a nomogram model (Fig. 6C). The calibration curves confirmed that the predicted overall survival at 1, 3, and 5 years using the nomogram model fit well with the actual values (Fig. 6D). In the KM curve, patients in the HRP group (as predicted by the nomogram model) also showed a significantly unfavorable prognosis compared to those in the LRP group (Fig. 6E). Additionally, the AUC of the 1-, 3-, and 5-year receiver operating characteristic curves were 0.761, 0.739, and 0.703, respectively, confirming the predictive reliability of the nomogram model (Fig. 6F).

### 3.7. Associations between prognostic risk with HCC subtypes and drug sensitivity

**Figure 7.**
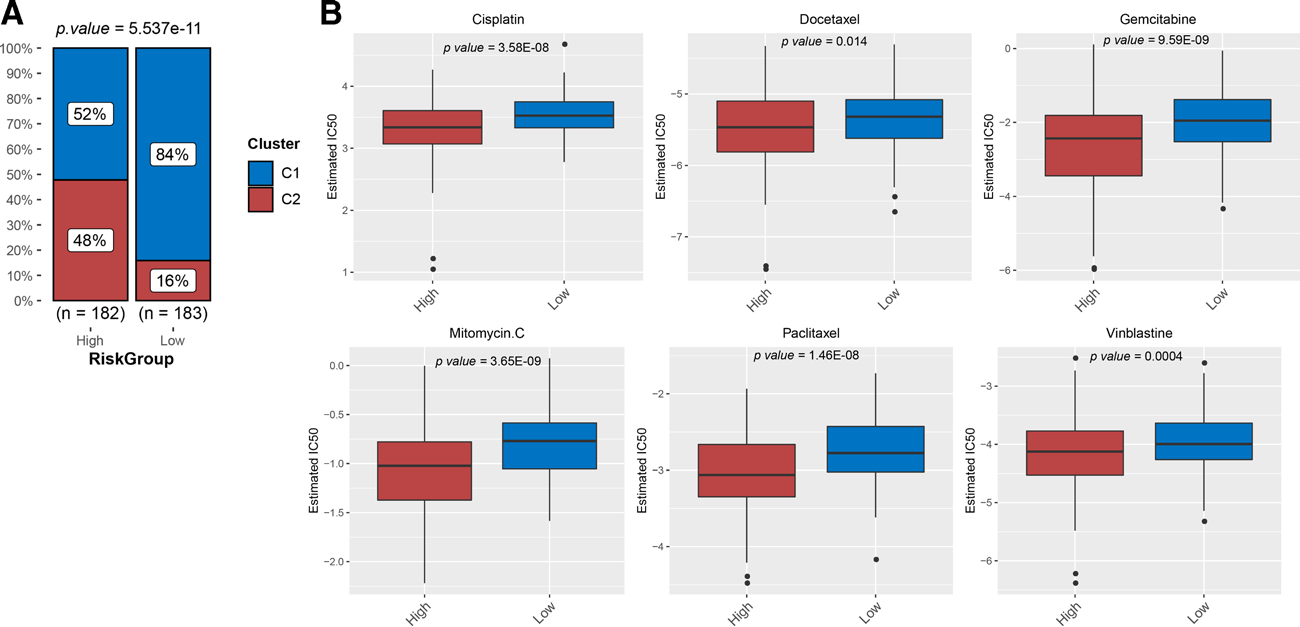
Difference of distribution of the 2 clusters and comparative drug sensitivity between HRP and LRP groups. (A) Comparison of the distributions of 2 clusters between the HRP and LRP groups. (B) Box plots showing differences between the 2 groups in IC50 values of 6 frequently used chemotherapy drugs for HCC. HCC = hepatocellular carcinoma, HRP = high-risk prognosis, IC50 = 50% inhibitory concentration, LRP = low-risk prognosis.

To observe the relationship between the HCC subtypes and risk groups, we compared the distribution of the 2 clusters between the HRP and LRP groups. The IC50 values of all drugs were significantly higher in the LRP group than in the HRP group (Fig. 7A), indicating that the high-risk group has higher drug sensitivities. Finally, 64 of the 138 chemotherapeutic drugs showed significantly different IC50 values between the HRP and LRP groups. Using box plots (Fig. 7B), we listed the differences in IC50 values between the 2 groups for the 6 chemotherapy drugs frequently used for HCC. The results showed that the IC50 values of these drugs were lower in the high-risk group than in the lower-risk group, indicating that differences in drug sensitivity may be a source of prognostic risk for patients with HCC.

## 4. Discussion

In this study, 16 of the 28 TRP channel-related genes were differentially expressed between HCC and non-tumorous sample tissues. Based on these key TRP channel-related genes, we identified 2 molecular HCC subtypes, of which Cluster 1 had a higher TRP score and possibly a more favorable prognosis. Analysis of prognostic factors and clinical features confirmed the prognostic characteristics of Cluster 1. Compared with patients in Cluster 2, more patients in Cluster 1 were distributed in the early stage of HCC and were characterized by a low malignant level of the disease. After screening the DEGs between Clusters 1 and 2 and constructing the prognostic model, we found that prognostic signatures based on the HCC subtype could predict prognostic risks effectively. Moreover, patients in Cluster 1 were more likely to be distributed in the low-risk group than those in Cluster 2. These results corroborate each other, demonstrating the reliability of subtype-based clustering and the successful construction of the prognostic model and confirming the important role of the 16 TRP channel-related genes in predicting the prognosis of HCC. Taken together, the prognostic signatures related to TRP channel genes and molecular subtypes obtained in this study can be used in clinical applications to predict HCC risk.

Identification of the immune microenvironment characteristics of the 2 subtypes revealed that the most significant difference between Clusters 1 and 2 was in the infiltration levels of M1 macrophages. Macrophages play a vital role in many pathophysiological processes such as inflammation, tissue repair, and metabolism. The polarization of M1/M2 macrophages may affect immune escape, carcinogenesis, and metastasis in HCC^[31,32]^ However, in our study, no difference between clusters was detected in terms of the infiltration level of M2 macrophages. However, a relevant study identified DEGs between high- and low-infiltrating M1 macrophages as predictors of HCC prognosis^[33]^ The activation of Notch signaling in HCC can mediate the differentiation of macrophages into M1 macrophages, thereby promoting inflammation and anti-tumor activity.^[34]^ In this study, a significantly increased infiltration of M1 macrophages was observed in Cluster 1, indicating the activation of the inflammatory response and anti-tumor activity against HCC. This is why patients in Cluster 1 showed better clinical outcomes. M1 macrophages play an anti-tumor role by inhibiting tumor cell proliferation and metastasis,^[35]^ which is consistent with our findings. Another finding is that the activation of the TRP family-related gene, *TRPC5* (which in this study was highly expressed in paracarcinoma tissues), may inhibit macrophage differentiation by regulating the Akt/IκB/NF-κB signaling pathway.^[36]^ Therefore, we further speculated that *TRPC5* could cause increased infiltration of M1 macrophages, thereby reducing the invasiveness of tumor cells, inhibiting their malignant phenotype, and promoting a favorable prognosis for patients with HCC. However, the regulatory mechanism of M1 macrophages and the role of M1/M2 polarization in HCC remain to be experimentally explored.

Considering the differences in overall survival and clinical phenotypes between the 2 HCC subtypes, we further analyzed the DEGs to explore the underlying molecular regulatory differences between Clusters 1 and 2 and screened prognostic signatures for the construction of a prognostic model and prognostic risk prediction. Among these, *FTCD, PPARGC1A, OGN, BRSK2, SPP1*, and *MMP1* have important prognostic values and can be used to define prognostic risks. Specifically, *FTCD, PPARGC1A*, and *OGN* are protective factors against HCC and are associated with a better prognosis. Chen et al confirmed our findings and proposed that low expression of *FTCD* is associated with poor prognosis and an aggressive tumor phenotype in HCC.^[37]^ The sensitivity and specificity of *FTCD* expression levels in differentiating normal or liver cirrhosis from early well-differentiated HCCs are 90% and 86.7%, respectively.^[38]^ Conversely, *BRSK2, SPP1*, and *MMP1* expression levels were associated with a higher risk of poor prognosis in this study. Li et al further demonstrated that *BRSK2* is positively associated with cancer status, has a prognostic risk for HCC, and may be involved in the regulatory mechanism of m6A methylation.^[39]^ *SPP1* is also a signature gene in HCC tissues that enhances the proliferation of tumor cells and is closely related to tumor progression.^[40]^ More importantly, *SPP1* mediates the crosstalk between HCC cells and macrophages and triggers the polarization of macrophages towards the M2 phenotype.^[41]^ However, the association between these genes and the TRP family has rarely been studied, except for *MMP1. MMP1* is upregulated in patients with HCC and is associated with a poor prognosis.^[42]^ Pharmacological inhibition and gene silencing of *TRPA1* downregulates MMP1 production in osteoarthritis,^[43]^ whereas activation of *TRPV1* increases the expression of *MMP1*.^[44]^ However, in the present study, specific levels of *TRPA1* and *TRPV1* were not detected in HCC tissues. Therefore, the relationship between the expression of these prognostic gene signatures and the TRP channel genes requires further confirmation.

There were several limitations in this study. First, there was no analysis of the potential associations between key TRP genes, M1 macrophage infiltration, and prognostic signatures. Second, no experimental verification of the diagnostic and prognostic values of key genes was performed. Third, all analysis was based on public databases, and no functional assay was conducted. Hence, in subsequent studies, we will focus on the interaction between M1/M2 macrophage polarization and TRP channel signaling, as well as on their actual impact on the prognosis of HCC.

## 5. Conclusion

In this study, 2 HCC subtypes were identified based on key TRP channel-related genes. Patients in Cluster 1 were prone to a favorable prognosis and a lower level of tumor malignancy. Based on differential gene screening between Clusters 1 and 2, and prognostic signature identification, we constructed prognostic and nomogram models to predict and stratify the prognostic risks of HCC. The validation results confirm the robust prediction performance of the models. However, subsequent experimental verification is required.

## Author contributions

**Conceptualization:** Jianxing Zheng.

Data curation: Dongyang Wu.

Formal analysis: Qingshan Cai.

Investigation: Dong Liu.

Methodology: Ganggang Zuo.

Resources: Shudong Li.

Validation: Liyou Liu.

## References

[1] Harris PS Hansen RM Gray ME. Hepatocellular carcinoma surveillance: an evidence-based approach. World J Gastroenterol. 2019;25:1550–9. 30983815 10.3748/wjg.v25.i13.1550 PMC6452232

[2] Wege H Schulze K von Felden J. Rare variants of primary liver cancer: fibrolamellar, combined, and sarcomatoid hepatocellular carcinomas. Eur J Med Genet. 2021;64:104313104313. 10.1016/j.ejmg.2021.104313 34418585

[3] Villanueva A. Hepatocellular carcinoma. N Engl J Med. 2019;380:1450–62. 30970190 10.1056/NEJMra1713263

[4] Frenette C. Advances in hepatocellular carcinoma. Clin Liver Dis. 2020;24: xiii–xiv. 10.1016/j.cld.2020.08.014 33012459

[5] Facciorusso A. The influence of diabetes in the pathogenesis and the clinical course of hepatocellular carcinoma: recent findings and new perspectives. Curr Diabetes Rev. 2013;9:382–6. 23845075 10.2174/15733998113099990068

[6] Facciorusso A Abd El Aziz M Singh S. Statin use decreases the incidence of hepatocellular carcinoma: an updated meta-analysis. Cancers (Basel). 2020;12:874. 32260179 10.3390/cancers12040874 PMC7225931

[7] Sugawara Y Hibi T. Surgical treatment of hepatocellular carcinoma. Biosci Trends. 2021;15:138–41. 33746184 10.5582/bst.2021.01094

[8] Heller M Parikh ND Fidelman N. Frontiers of therapy for hepatocellular carcinoma. Abdom Radiol (NY). 2021;46:3648–59. 33837453 10.1007/s00261-021-03065-0

[9] Facciorusso A Abd El Aziz M Sacco R. Efficacy of regorafenib in hepatocellular carcinoma patients: a systematic review and meta-analysis. Cancers. 2019;12:36. 31877664 10.3390/cancers12010036 PMC7017079

[10] Garuti F Neri A Avanzato F. The changing scenario of hepatocellular carcinoma in Italy: an update. Liver Int. 2021;41:585–97. 33219585 10.1111/liv.14735

[11] Chidambaranathan-Reghupaty S Fisher PB Sarkar D. Hepatocellular carcinoma (HCC): epidemiology, etiology and molecular classification. Adv Cancer Res. 2021;149:1–61. 33579421 10.1016/bs.acr.2020.10.001 PMC8796122

[12] Maennich D Marshal L. Hepatocellular carcinoma. Nat Rev Dis Primers. 2021;7:7. 33479233 10.1038/s41572-021-00245-6

[13] Samanta A Hughes TET Moiseenkova-Bell VY. Transient receptor potential (TRP) channels. Subcell Biochem. 2018;87:141–65. 29464560 10.1007/978-981-10-7757-9_6 PMC6038138

[14] Asghar MY Törnquist K. Transient receptor potential canonical (TRPC) channels as modulators of migration and invasion. Int J Mol Sci . 2020;21:1739. 32138386 10.3390/ijms21051739 PMC7084769

[15] Gao YY Tian W Zhang HN. Canonical transient receptor potential channels and their modulators: biology, pharmacology and therapeutic potentials. Arch Pharm Res. 2021;44:354–77. 33763843 10.1007/s12272-021-01319-5 PMC7989688

[16] Yang D Kim J. Emerging role of transient receptor potential (TRP) channels in cancer progression. BMB Rep. 2020;53:125–32. 32172727 10.5483/BMBRep.2020.53.3.016 PMC7118349

[17] Siveen KS Nizamuddin PB Uddin S. TRPV2: A cancer biomarker and potential therapeutic target. Dis Markers. 2020;2020:8892312. 33376561 10.1155/2020/8892312 PMC7746447

[18] Hu Z Cao X Fang Y. Transient receptor potential vanilloid-type 2 targeting on stemness in liver cancer. Biomed Pharmacother 2018;105:697–706. 29906748 10.1016/j.biopha.2018.06.029

[19] Liu G Xie C Sun F. Clinical significance of transient receptor potential vanilloid 2 expression in human hepatocellular carcinoma. Cancer Genet Cytogenet. 2010;197:54–9. 20113837 10.1016/j.cancergencyto.2009.08.007

[20] Zhu S Ye H Xu X. Involvement of TRPC7-AS1 expression in hepatitis B virus-related hepatocellular carcinoma. J Oncol. 2021;2021:8114327. 34512754 10.1155/2021/8114327 PMC8426092

[21] Wang C Liao Y He W. Elafin promotes tumour metastasis and attenuates the anti-metastatic effects of erlotinib via binding to EGFR in hepatocellular carcinoma. J Exp Clin Cancer Res 2021;40:113. 33771199 10.1186/s13046-021-01904-y PMC7995733

[22] Pan F Wang K Zheng M. A TRP family based signature for prognosis prediction in head and neck squamous cell carcinoma. J Oncol. 2022;2022:8757656. 35140788 10.1155/2022/8757656 PMC8820906

[23] Hänzelmann S Castelo R Guinney J. GSVA: gene set variation analysis for microarray and RNA-seq data. BMC Bioinf. 2013;14:7. 10.1186/1471-2105-14-7 PMC3618321 23323831

[24] Rizvi AA Karaesmen E Morgan M. gwasurvivr: an R package for genome-wide survival analysis. Bioinformatics. 2019;35:1968–70. 30395168 10.1093/bioinformatics/bty920 PMC7963072

[25] Chen B Khodadoust MS Liu CL. Profiling tumor infiltrating immune cells with CIBERSORT. Methods Mol Biol. 2018;1711:243–59. 29344893 10.1007/978-1-4939-7493-1_12 PMC5895181

[26] Hu D Zhou M Zhu X. Deciphering immune-associated genes to predict survival in clear cell renal cell cancer. Biomed Res Int. 2019;2019:2506843. 31886185 10.1155/2019/2506843 PMC6925759

[27] Ritchie ME Phipson B Wu D. limma powers differential expression analyses for RNA-sequencing and microarray studies. Nucleic Acids Res. 2015;43:e47. 25605792 10.1093/nar/gkv007 PMC4402510

[28] Zhang S Tong YX Zhang XH. A novel and validated nomogram to predict overall survival for gastric neuroendocrine neoplasms. J Cancer. 2019;10:5944–54. 31762804 10.7150/jca.35785 PMC6856574

[29] Geeleher P Cox N Huang RS. pRRophetic: an R package for prediction of clinical chemotherapeutic response from tumor gene expression levels. PLoS One. 2014;9:e107468. 25229481 10.1371/journal.pone.0107468 PMC4167990

[30] Brunson J. ggalluvial: layered grammar for alluvial plots. J Open Source Softw. 2020;5:2017. 10.21105/joss.02017 PMC10010671 36919162

[31] Yin Z Ma T Lin Y. IL-6/STAT3 pathway intermediates M1/M2 macrophage polarization during the development of hepatocellular carcinoma. J Cell Biochem. 2018;119:9419–32. 30015355 10.1002/jcb.27259

[32] Ye Y Xu Y Lai Y. Long non-coding RNA cox-2 prevents immune evasion and metastasis of hepatocellular carcinoma by altering M1/M2 macrophage polarization. J Cell Biochem. 2018;119:2951–63. 29131381 10.1002/jcb.26509

[33] Zhang H Sun L Hu X. Macrophages M1-related prognostic signature in hepatocellular carcinoma. J Oncol. 2021;2021:6347592. 34745260 10.1155/2021/6347592 PMC8486543

[34] Chen W Liu Y Chen J. The Notch signaling pathway regulates macrophage polarization in liver diseases. Int Immunopharmacol. 2021;99:107938. 34371331 10.1016/j.intimp.2021.107938

[35] Wang C Ma C Gong L. Macrophage polarization and its role in liver disease. Front Immunol. 2021;12:803037. 34970275 10.3389/fimmu.2021.803037 PMC8712501

[36] Tao L Guo G Qi Y. Inhibition of canonical transient receptor potential 5 channels polarizes macrophages to an M1 phenotype. Pharmacology. 2020;105:202–8. 31618743 10.1159/000503452

[37] Chen J Chen Z Huang Z. Formiminotransferase cyclodeaminase suppresses hepatocellular carcinoma by modulating cell apoptosis, DNA damage, and phosphatidylinositol 3-kinases (PI3K)/Akt signaling pathway. Med Sci Monit. 2019;25:4474–84. 31203308 10.12659/MSM.916202 PMC6592141

[38] Labib OH Harb OA Khalil OH. The diagnostic value of Arginase-1, FTCD, and MOC-31 expression in early detection of hepatocellular carcinoma (HCC) and in differentiation between HCC and metastatic adenocarcinoma to the liver. J Gastrointest Cancer. 2020;51:88–101. 30784016 10.1007/s12029-019-00211-2

[39] Li Y Qi D Zhu B. Analysis of m6A RNA methylation-related genes in liver hepatocellular carcinoma and their correlation with survival. Int J Mol Sci. 2021;22:1474. 33540684 10.3390/ijms22031474 PMC7867233

[40] Wang J Hao F Fei X. SPP1 functions as an enhancer of cell growth in hepatocellular carcinoma targeted by miR-181c. Am J Transl Res. 2019;11:6924–37. 31814897 PMC6895505

[41] Liu L Zhang R Deng J. Construction of TME and identification of crosstalk between malignant cells and macrophages by SPP1 in hepatocellular carcinoma. Cancer Immunol Immunother. 2022;71:121–36. 34028567 10.1007/s00262-021-02967-8 PMC10992184

[42] Dai L Mugaanyi J Cai X. Comprehensive bioinformatic analysis of MMP1 in hepatocellular carcinoma and establishment of relevant prognostic model. Sci Rep. 2022;12:13639. 35948625 10.1038/s41598-022-17954-x PMC9365786

[43] Yin S Wang P Xing R. Transient receptor potential Ankyrin 1 (TRPA1) mediates lipopolysaccharide (LPS)-induced inflammatory responses in primary human osteoarthritic fibroblast-like synoviocytes. Inflammation. 2018;41:700–9. 29318481 10.1007/s10753-017-0724-0

[44] Li WH Lee YM Kim JY. Transient receptor potential vanilloid-1 mediates heat-shock-induced matrix metalloproteinase-1 expression in human epidermal keratinocytes. J Invest Dermatol. 2007;127:2328–35. 17508023 10.1038/sj.jid.5700880

